# The potential impact of media reporting in syndromic surveillance: an example using a possible C*ryptosporidium* exposure in North West England, August to September 2015

**DOI:** 10.2807/1560-7917.ES.2016.21.41.30368

**Published:** 2016-10-13

**Authors:** Alex J Elliot, Helen E Hughes, John Astbury, Grainne Nixon, Kate Brierley, Roberto Vivancos, Thomas Inns, Valerie Decraene, Katherine Platt, Iain Lake, Sarah J O’Brien, Gillian E Smith

**Affiliations:** 1 Public Health England, Birmingham, United Kingdom; 2 NIHR HPRU in Emergency Preparedness and Response, London, United Kingdom; 3 Farr Institute at HeRC, Liverpool, United Kingdom; 4 NIHR HPRU in Gastrointestinal Infections, Liverpool, United Kingdom; 5 Public Health England, Preston, United Kingdom; 6 Public Health England, Liverpool, United Kingdom; 7 NIHR HPRU in Emerging and Zoonotic Infections, Liverpool, United Kingdom; 8 University of East Anglia, Norwich, United Kingdom

**Keywords:** syndromic surveillance, *Cryptosporidium*, diarrhoea, gastroenteritis, media, bias

## Abstract

During August 2015, a boil water notice (BWN) was issued across parts of North West England following the detection of *Cryptosporidium* oocysts in the public water supply. Using prospective syndromic surveillance, we detected statistically significant increases in the presentation of cases of gastroenteritis and diarrhoea to general practitioner services and related calls to the national health telephone advice service in those areas affected by the BWN. In the affected areas, average in-hours general practitioner consultations for gastroenteritis increased by 24.8% (from 13.49 to 16.84) during the BWN period; average diarrhoea consultations increased by 28.5% (from 8.33 to 10.71). Local public health investigations revealed no laboratory reported cases confirmed as being associated with the water supply. These findings suggest that the increases reported by syndromic surveillance of cases of gastroenteritis and diarrhoea likely resulted from changes in healthcare seeking behaviour driven by the intense local and national media coverage of the potential health risks during the event. This study has further highlighted the potential for media-driven bias in syndromic surveillance, and the challenges in disentangling true increases in community infection from those driven by media reporting.

## Introduction

Since its first identification as a cause of human infection, the protozoan parasite *Cryptosporidium* has been established as a significant cause of morbidity and mortality globally [1]. Over 20 different *Cryptosporidium* species have been recognised, with 15 currently reported to cause human infection. However the majority of human infections are associated with infection from *Cryptosporidium hominis* and *Cryptosporidium parvum* [2]. Cryptosporidiosis is particularly associated with prolonged and persistent diarrhoea, however it is also characterised by abdominal pain, nausea and/or vomiting [3,4]. Transmission is through the faecal–oral route; symptoms generally occur between 2 to 12 days post infection with a mean incubation period of 5 to 7 days. The burden of *Cryptosporidium* is greater in children and those who are malnourished or immunocompromised [5,6].

In high income countries, *Cryptosporidium* is a leading cause of waterborne outbreaks. One of the largest and best described outbreaks occurred in Milwaukee (Wisconsin, United States) during 1993, where over 400,000 people using a municipal water supply were affected during a two month period [7]. In England, recreational water *Cryptosporidium* outbreaks, e.g. associated with swimming pools, are far more common than those involving public drinking water supplies [8]. Four previous drinking water outbreaks have been described in England, including the largest in the East Midlands where contamination of the local water supply resulted in an estimated 400 excess cases of diarrhoea and 23 laboratory-confirmed cases [9]. As the detection of oocysts in water samples can indicate a potential risk to health, the water supplier may decide to issue a boil water notice (BWN), advising the affected populations to boil all water before drinking [10]. In previous studies evaluating the public’s understanding and compliance with BWNs, varying levels of compliance during the notice period were revealed [11-15]. In England the decision to lift a BWN is taken by the water supplier, in consultation with public health organisations.

In England, during any incident where *Cryptosporidium* oocysts have been detected in a public water supply, a number of different public health surveillance systems, including laboratory reporting and syndromic surveillance, are used to identify the impact, if any, on disease burden. Syndromic surveillance can be used both to assess increases in the healthcare consultations e.g. to primary care, and to reassure lack of impact where there are no changes detected in healthcare seeking behaviour.

Between 31 July and 4 August 2015 *Cryptosporidium* oocysts were identified in a water treatment works supplying drinking water to parts of the North West England region. As a result a BWN was issued on 6 August 2015 in the areas concerned. We describe the use of syndromic surveillance to monitor healthcare seeking behaviour in those areas affected, to determine whether increases in the presentation of gastroenteritis symptoms were linked to the alert.

### Cryptosporidium alert

**Figure 1:**
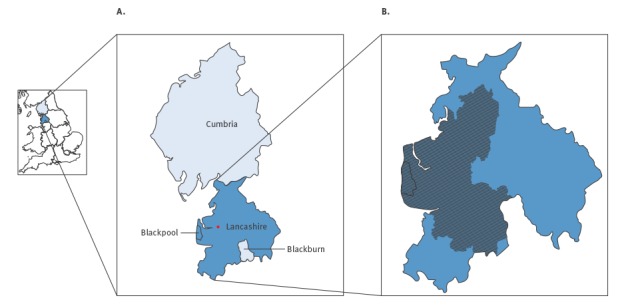
Location of Blackpool and Lancashire upper tier local authorities (LAs) as well as the postcode districts in these two LAs, which were affected by a boil water notice, North West England, 6 August−6 September 2015

Routine testing of water supplies at Franklaw water treatment works (which supplied drinking water to the affected areas), detected low numbers of *Cryptosporidium* oocysts between 31 July and 4 August 2015 (initial sample results of 0.031 and 0.119 oocysts per 10 L water were well below 0.2 oocysts per 10 L, the ‘trigger’ level where measures such as flushing the water network or closing the plant become necessary). A BWN was issued on 6 August across Lancashire and Blackpool upper tier local authorities (LAs: across England local government functions are divided between two tiers of local authority, upper and lower tier local authority), affecting ca 300,000 households and attracting local media coverage (Figure 1). Water samples taken across the affected water network remained positive for *Cryptosporidium* over the next few weeks, albeit below the ‘trigger’ level. To clear the system of *Cryptosporidium*, the water authority adopted a combination of flushing the water network, transferring water from other parts of the network and installing ultraviolet light rigs. It was decided that before the BWN could be lifted in any given part of the network supplied by the Franklaw water plant, water sampling should be negative on three consecutive days. Across various parts of the network, as negative samples were identified, the BWN was lifted: on 27 August the BWN was partially lifted across parts of Blackpool; over the next 10 days the BWN was gradually lifted across further areas, until 6 September, when the BWN was lifted across the whole water network. The routine local public health investigation revealed that there were no laboratory reported cases which could be confirmed to be associated with the water supply either before, during or after the BWN (data now shown).

(A) The area of the North West England region, covered by the Cumbria and Lancashire Public Health England (PHE) team (which includes Blackburn, Blackpool, Cumbria and Lancashire LAs), is coloured in different tones of blue. Within this area, Blackpool and Lancashire LAs, which were affected by the boil water notice are in darker blue. The location of the affected water treatment works is illustrated by a red circle.

(B) Postcode districts affected by the boil water notice within Blackpool and Lancashire LAs are shaded.

## Methods

### Syndromic surveillance

Syndromic surveillance is the near real-time collection, analysis, interpretation and dissemination of health-related data to enable the early identification of the impact (or absence of impact) of potential human or veterinary public-health threats which require effective public health action [16]. The Public Health England (PHE) Real-time Syndromic Surveillance Team (ReSST) coordinates a suite of national syndromic surveillance systems and delivers a real-time syndromic surveillance service that has been described in detail elsewhere [17]. In brief, daily data are collected from a number of healthcare provider sources and analysed, interpreted and risk assessed using statistical algorithms (modelling historical data to identify significant increases in activity) [18]. The data received are aggregated into a number of syndromic indicators based upon symptoms and clinical diagnosis of disease.

For this incident, telehealth (National Health Service (NHS) telephone advice, NHS 111) calls, general practitioner (GP) in-hours (GP IH) and GP out-of-hours (GP OOH) syndromic surveillance data for gastroenteritis, diarrhoea and vomiting were used. NHS 111 calls were based upon such symptoms reported by patients, while GP consultations included those where the clinical diagnosis made by the GP involved clinical codes relating to gastroenteritis, diarrhoea or vomiting. The population coverage of each system in the LAs issued with the BWN and those neighbouring the BWN area was initially assessed to ensure that there was sufficient surveillance coverage: GP OOH coverage in Blackburn LA (which neighboured the LAs with the BWN) was insufficient for surveillance and, therefore, was not included in the results.

### Epidemiological analysis

NHS 111 telephone calls, GP IH and GP OOH syndromic surveillance data were monitored during the period of the BWN (6 August to 5 September) and for 14 days after. Daily data counts were plotted as rates per 100,000 population (GP IH) and per cent of indicator to total calls/consultations (NHS 111/GP OOH) with 3 day moving averages included to aid interpretation. Data were analysed by LA, including two which were affected by the BWN (Blackpool LA and Lancashire LA) and two neighbouring LAs not affected by the BWN (Blackburn LA and Cumbria LA). Data were also analysed for the Cumbria and Lancashire PHE local health protection team area [19], which included a footprint covering all four LAs (Figure 1).

### Statistical analysis

Routine statistical analysis of syndromic surveillance data was undertaken prospectively on a daily basis during the study period using automated statistical models to identify significant exceedances compared with either recent activity, or historically expected levels. The routine statistical methods used are described in detail elsewhere however in summary a baseline was estimated for each system and syndromic indicator using a multi-level hierarchical mixed effects model incorporating appropriate variables (e.g. day of the week and public holidays) [18]. An upper 99% prediction interval threshold for expected activity each day was established using the estimated baselines, adjusting for variation in the total volume of daily data received. Exceedances were assessed as significant where the actual number of consultations or calls exceeded these 99% prediction interval thresholds [18].

A Student’s two-tailed test was used to determine differences in the mean syndromic surveillance daily data during the BWN (6 August to 5 September) and a comparative period of 31 days (2 July to 1 August; the same sequence and number of days as the BWN were included) preceding the BWN (‘non-BWN’ period). Weekends (when GP IH services are closed) were removed from the analysis of GP IH data resulting in comparative periods of 21 days. A mean of the daily syndromic surveillance data was taken for each geographical location and syndromic indicator separately, for the period of the BWN. Results for Blackpool and Lancashire LAs were compared with two neighbouring LAs not issued with the BWN (Blackburn and Cumbria LAs), Cumbria and Lancashire PHE team area, as well as England.

All statistical analyses were undertaken using Stata v13 [20].

## Results

### Epidemiological analysis

**Figure 2:**
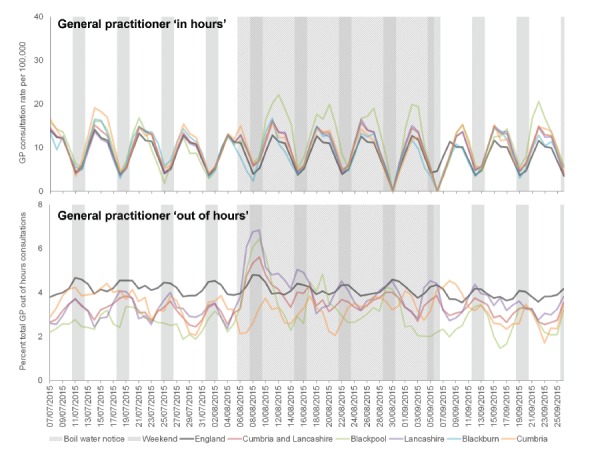
Daily presentation (3 day moving average) of gastroenteritis consultations to general practitioner services (in-hours and out-of-hours) in North West England, 7 July−26 September 2015

There was an apparent increase in GP consultations for gastroenteritis during the period of the BWN in the two affected LAs. GP OOH consultations increased immediately following the issue of the BWN, with the highest peak occurring in Lancashire LA. The peak in GP IH consultations occurred a few days later (following a weekend), and peaked highest in Blackpool LA (Figure 2). The increases in the two affected LAs were reflected at the level of the PHE team area of Cumbria and Lancashire, where GP IH consultation rates for gastroenteritis remained at slightly elevated levels for the duration of the BWN, before subsequently returning to expected levels.

Data presented for England (dark green), Cumbria and Lancashire Public Health England (PHE) team area (brown) and for upper tier local authorities (LAs) within this team area (Blackburn, light blue; Blackpool, light green; Cumbria, light orange and Lancashire, purple).

**Figure 3:**
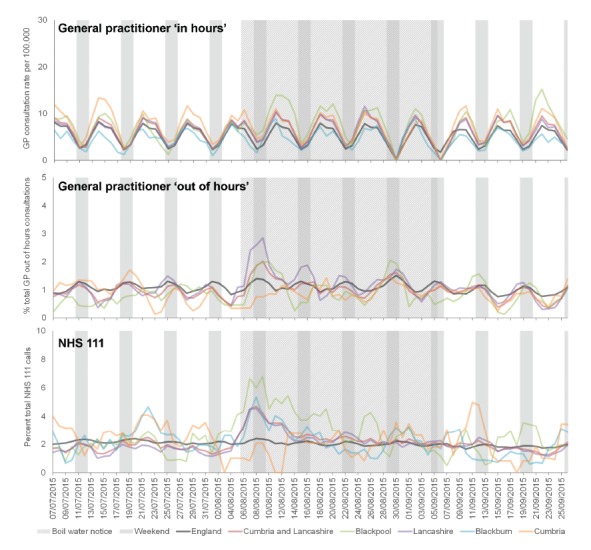
Daily presentation (3 day moving average) of diarrhoea general practitioner (GP) consultations and National Health Service (NHS) 111 calls in North West England, 7 July−26 September 2015

GP OOH consultations for diarrhoea increased immediately following the BWN, and peaked before GP IH diarrhoea consultations; Lancashire LA peaked highest in the GP OOH and Blackpool LA in the GP IH (Figure 3). NHS 111 calls for diarrhoea peaked concurrently with GP OOH and peaked highest in Blackpool LA. GP IH consultation rates for diarrhoea remained at elevated levels for the duration of the BWN, before returning to expected levels once the BWN was lifted.

Data presented for England (dark green), Cumbria and Lancashire Public Health England (PHE) team area (brown) and for upper tier local authorities (LAs) within this team area (Blackburn, light blue; Blackpool, light green; Cumbria, light orange and Lancashire, purple).

**Figure 4:**
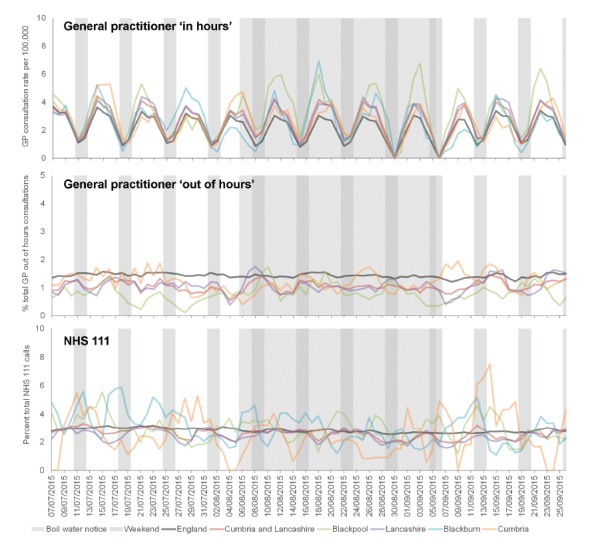
Daily presentation (3 day moving average) of vomiting general practitioner (GP) consultations and National Health Service (NHS) 111 calls in North West England, 7 July−26 September 2015

GP IH consultations for vomiting showed a similar increase during the BWN period however this was only noted in Blackpool LA. There were no increases in vomiting presentations in the GP OOH or NHS 111 systems (Figure 4).

Data presented for England (dark green), Cumbria and Lancashire Public Health England (PHE) team area (brown) and for upper tier local authorities (LAs) within this team area (Blackburn, light blue; Blackpool, light green; Cumbria, light orange and Lancashire, purple).

### Routine statistical analysis

Routine statistical analysis of the data received by ReSST on a daily basis illustrated significant increases in the gastroenteritis and diarrhoea indicators at the LA level, occurring on the day of, and immediately following the issue of the BWN (Table 1). The frequency of the statistically significant alarms decreased after 9 August, after which few alarms occurred.

**Table 1:** Routine analyses resulting in statistical alarms for syndromic surveillance systems in Blackpool and Lancashire upper tier local authorities (LA), the two LAs affected by the boil water notice, North West England, 1 August−16 September 2015

System	Diarrhoea						Gastroenteritis				Vomiting					
	Blackpool			Lancashire			Blackpool		Lancashire		Blackpool			Lancashire		
	NHS 111	GP IH	GP OOH	NHS 111	GP IH	GP OOH	GP IH	GP OOH	GP IH	GP OOH	NHS 111	GP IH	GP OOH	NHS 111	GP IH	GP OOH
01/8/15	N	NA	N	N	NA	N	NA	N	NA	N	N	NA	N	N	NA	N
02/8/15	N	NA	N	N	NA	N	NA	N	NA	N	N	NA	N	N	NA	N
03/8/15	N	N	N	N	N	N	N	N	N	N	N	N	N	N	N	N
04/8/15	N	N	N	N	N	N	N	N	N	N	N	N	N	N	N	N
05/8/15	N	N	N	N	N	N	N	N	N	N	N	N	N	N	N	N
06/8/15^a^	Y	N	N	Y	N	Y	N	N	N	Y	N	N	N	N	N	N
07/8/15	N	Y	N	Y	Y	N	Y	N	Y	N	N	N	N	N	Y	Y
08/8/15	Y	NA	Y	Y	NA	Y	NA	Y	NA	Y	N	NA	N	N	NA	Y
09/8/15	N	NA	Y	Y	NA	Y	NA	Y	NA	Y	N	NA	Y	N	NA	Y
10/8/15	N	Y	N	N	N	Y	N	N	Y	N	N	N	Y	N	N	N
11/8/15	N	N	Y	N	N	N	Y	N	N	N	N	N	N	N	N	N
12/8/15	N	N	N	N	N	N	N	N	N	N	N	N	N	N	N	N
13/8/15	N	Y	N	N	N	N	Y	N	N	N	N	N	N	N	N	N
14/8/15	N	N	N	N	N	N	N	N	N	N	N	N	N	N	N	N
15/8/15	N	NA	N	N	NA	N	NA	N	NA	N	N	NA	N	N	NA	N
16/8/15	N	NA	N	N	NA	N	NA	N	NA	Y	N	NA	N	N	NA	N
17/8/15	N	N	N	N	N	N	N	N	N	N	N	N	N	N	N	N
18/8/15	N	N	Y	N	N	N	N	Y	Y	N	N	N	N	N	N	N
19/8/15	N	N	N	N	N	N	N	N	N	N	N	N	N	N	N	N
20/8/15	N	N	N	N	N	N	N	N	N	N	N	N	Y	N	N	N
21/8/15	N	N	N	N	N	N	Y	N	N	N	N	N	N	N	N	N
22/8/15	N	NA	N	N	NA	N	N	N	NA	N	N	NA	N	N	NA	N
23/8/15	N	NA	N	N	NA	N	N	N	NA	N	N	NA	N	N	NA	N
24/8/15	N	N	N	N	N	N	N	N	N	N	N	N	N	N	N	N
25/8/15	N	N	N	N	N	N	N	N	Y	N	N	N	N	N	N	N
26/8/15	Y	N	N	N	N	N	N	N	Y	N	N	N	N	N	N	N
27/8/15^b^	N	N	N	N	N	N	N	N	N	N	N	N	N	N	N	N
28/8/15	N	N	N	N	N	N	Y	N	N	N	N	N	N	N	N	N
29/8/15	N	NA	N	N	NA	N	NA	N	NA	N	N	NA	N	N	NA	N
30/8/15	N	NA	Y	N	NA	N	NA	Y	NA	N	N	NA	N	N	NA	Y
31/8/15	N	NA	N	N	NA	N	NA	N	NA	N	N	NA	N	N	NA	N
01/9/15	N	N	N	N	N	N	N	N	N	N	N	N	N	N	N	N
02/9/15	N	N	N	N	N	N	Y	N	N	N	N	N	N	N	N	N
03/9/15	N	N	N	N	N	N	N	N	N	N	N	N	N	N	N	N
04/9/15	N	N	N	N	N	N	N	N	Y	N	N	N	N	N	N	N
05/9/15	N	NA	N	Y	NA	N	NA	N	NA	Y	N	NA	N	N	NA	N
06/9/15^c^	N	NA	N	N	NA	N	NA	N	NA	N	N	NA	N	N	NA	N
07/9/15	N	N	N	N	N	N	N	N	Y	N	N	N	N	N	N	N
08/9/15	N	N	N	N	N	N	N	N	N	N	N	N	N	N	N	N
09/9/15	N	N	N	N	N	N	N	N	N	N	N	N	N	N	N	N
10/9/15	N	N	N	N	N	N	N	N	N	N	N	N	N	N	N	N
11/9/15	N	N	N	N	N	N	N	N	N	N	Y	N	N	N	N	N
12/9/15	N	NA	N	N	NA	N	NA	N	NA	N	N	NA	N	Y	NA	Y
13/9/15	N	NA	N	N	NA	N	NA	Y	NA	N	N	NA	N	N	NA	N
14/9/15	N	N	N	N	N	N	N	N	N	N	N	N	N	N	N	N
15/9/15	N	N	N	N	N	N	N	N	N	N	N	N	N	N	N	Y
16/9/15	Y	N	N	N	N	N	N	N	N	N	N	N	N	N	N	N

GP IH: general practitioner in-hours system; GP OOH: general practitioner out-of-hours system; N: no statistically significant exceedance recorded; NA: not applicable (GP IH services not routinely available at weekends); NHS: National Health Service; Y: statistically significant exceedance recorded.

Dates are represented as day/month/year. Daily data are routinely collected from a number of healthcare provider sources and analysed, interpreted and risk assessed using automated statistical models (modelling historical data to identify significant increases in activity) [18]. Cells representing days with a statistically significant exceedance are highlighted in yellow. The issue and the partial or complete lifting of the boil water notice are indicated by dark blue shading. Weekends and public holidays are shaded in light blue.

^a^ Boil water notice.

^b^ Partial lifting of boil water notice.

^c^ Complete lifting of boil water notice across remaining areas.

Comparing syndromic surveillance data between the BWN (6 August – 5 September) and non-BWN (2 July – 1 August) periods revealed significant differences in those areas where the BWN had been issued (Table 2). Within Blackpool and Lancashire LAs GP IH gastroenteritis and diarrhoea mean consultation rates were significantly higher during the BWN (p < 0.01). Considering these two LAs together, the gastroenteritis GP IH average consultation rates during the BWN increased by 24.8% (i.e. from 13.49 to 16.84), while average diarrhoea consultations increased by 28.5% (8.33 to 10.71). In Blackpool LA, GP IH rates for gastroenteritis and diarrhoea were 33.5% and 35.4% higher during the BWN period while in Lancashire LA these were 15.2% and 20.8% higher. In the two neighbouring LAs not affected by the BWN, there were no significant differences observed at the 95% or 99% significance levels. At the PHE team area level (Cumbria and Lancashire), there were significant increases (p < 0.01) in gastroenteritis or diarrhoea across all systems. There were also significant results at the National (England) level, however these results were significant indicating higher incidence during the non-BWN period for selected indicators in the GP IH and NHS 111 systems. When comparing vomiting indicators across each system there were no significant differences between the BWN and non-BWN periods.

**Table 2:** Means of rates of general practitioner (GP) in-hours consultation, and percentages of GP out-of-hours consultations and National Health Service (NHS) 111 calls for gastroenteritis, diarrhoea and vomiting during the boil water notice and non-boil water notice periods, and comparison of the two periods, North West England, July−September 2015

System	Indicator and period		Blackpool LA		Lancashire LA		Blackburn LA		Cumbria LA		Cumbria and Lancashire^a^		England	
			Mean^b^	P value	Mean^b^	P value	Mean^b^	P value	Mean^b^	P value	Mean^b^	P value	Mean^b^	P value
GP IH	Gastroenteritis	BWN	18.954	**0.009**	14.728	**0.001**	12.545	0.103	14	0.098	14.68	**0.005**	12.069	0.002
		Non-BWN	14.2		12.786		14.401		15.240		13.526		12.732	
	Diarrhoea	BWN	11.951	**0.003**	9.462	**0.000**	6.931	0.449	9.786	0.107	9.472	**0.001**	7.424	0.17
		Non-BWN	8.829		7.835		6.331		10.825		8.375		7.553	
	Vomiting	BWN	5.258	0.036	3.823	0.328	3.939	0.789	3.719	0.384	3.914	0.286	2.856	0.000
		Non-BWN	3.754		3.642		4.069		3.983		3.758		3.289	
GP OOH	Gastroenteritis	BWN	3.531	**0.005**	4.343	**0.000**	NA	NA	3.157	0.142	3.839	**0.001**	4.165	0.457
		Non-BWN	2.586		3.245		NA		3.531		3.185		4.213	
	Diarrhoea	BWN	1.035	0.156	1.399	**0.004**	NA	NA	0.905	0.565	1.176	0.011	1.151	0.154
		Non-BWN	0.767		0.974		NA		0.982		0.943		1.101	
	Vomiting	BWN	0.922	0.08	1.090	0.891	NA	NA	1.083	0.178	1.06	0.903	1.418	0.136
		Non-BWN	0.658		1.079		NA		1.277		1.052		1.460	
NHS 111	Diarrhoea	BWN	3.647	**0.002**	2.626	**0.000**	2.487	0.946	1.883	0.05	2.695	**0.000**	2.068	0.046
		Non-BWN	2.126		1.627		2.462		3.338		1.864		2.154	
	Vomiting	BWN	2.897	0.68	2.453	0.394	2.756	0.059	2.058	0.121	2.509	0.018	2.738	0.000
		Non-BWN	3.034		2.596		3.767		3.008		2.817		2.966	

BWN: boil water notice period (6/8/15–8/9/15); GP IH: general practitioner in-hours system; GP OOH: general practitioner out-of-hours system; LA: local authority; NA: not applicable (GP OOH coverage in Blackburn LA was insufficient for surveillance and therefore not included in the results); non-BWN: non-boil water notice period (2/7/15–1/8/15).

P-values significant at the 99% level are highlighted bold where mean values were higher during the BWN period.

^a^ This refers to the Cumbria and Lancashire Public Health England area, which includes Blackburn, Blackpool, Cumbria and Lancashire LAs.

^b^ Mean of GP IH consultation rate, or GP OOH consultation percentage, or NHS 111 call percentage.

## Discussion

We present a description of the real-time monitoring of healthcare seeking behaviour using syndromic surveillance during a BWN following the detection of *Cryptosporidium* in the mains water supply to parts of North West England between 31 July and 4 August 2015. The BWN impacted on a large number of people (ca 300,000 households) in Blackpool and Lancashire LAs. Routine syndromic surveillance revealed significant increases in presentations to GPs (GP IH and GP OOH) and NHS 111 calls for diarrhoea and gastroenteritis in Blackpool and Lancashire LAs in the days immediately following the BWN. Rates of these indicators remained elevated for several days before returning to expected seasonal levels. There were no significant increases in neighbouring LAs where water supplies were unaffected. Interestingly, Lancashire LA was large in terms of geographical area (cf.d with Blackpool LA) however only certain areas of it were actually impacted by the BWN (Figure 1). This implied that the local impact in those areas affected was higher than that estimated for the LA as a whole.

Increases in GP OOH and NHS 111 indicators were observed immediately following the BWN whereas GP IH indicators peaked over the following days. The BWN was issued on a Thursday afternoon, meaning patients had more opportunity to access out of hours healthcare services, resulting in immediate increases compared with the routine GP services which patients were better able to access in the following week. This emphasises the importance of accessing syndromic surveillance data from a range of healthcare services, or those that are immediately available to the population, to accurately determine the peak of impact of an event.

As part of the local routine incident response, there were small increases in laboratory detections of *Cryptosporidium* identified from patient samples in Blackpool LA (data not available from other affected LAs) during week 35 (25–31 August 2015). In the affected area, laboratory reports increased from an average of one detection per week in the four preceding weeks to seven during week 35 and 12 during week 36, then falling to expected levels over the following two weeks. However, this coincided with a national increase of *Cryptosporidium* infection across England (peaking nationally week 37, 7–13 September 2015): there was also insufficient information to link individual cases within BWN areas to the local water supply, or there were other risk factors (e.g. history of travel) involved (data not shown). This, linked to the original low oocyst count in water samples suggested that it was highly likely that the increase in healthcare seeking behaviour monitored by syndromic surveillance during the BWN was due to intense local and national media reporting, rather than actual *Cryptosporidium* infections.

Local populations were informed of the BWN through printed and digital media and advised to seek medical advice if they had symptoms of cryptosporidiosis such as diarrhoea, including consulting a GP in order that faecal samples could be collected and tested to confirm *Cryptosporidium* infection. It is possible that this messaging therefore had several impacts: (i) symptomatic patients who would not normally have consulted a healthcare professional (i.e. they would have self-treated at home) would have been more likely to visit one of these services; (ii) the volume of tests requested would have increased possibly increasing the overall number of positive tests; (iii) healthcare professionals might have been more likely to notify cases or use more specific clinical codes relevant to infectious gastroenteritis based upon the knowledge of the BWN and the health implications. Other sources of data from the incident (data not shown) illustrated an increase in the volume of tests, where the average number of weekly laboratory tests for *Cryptosporidium* increased from an average of 155 per week in the four preceding weeks to 264 in both weeks 33 and 34 (10–23 August 2015), respectively, during the BWN period. However, during this peak in testing, positivity rates remained low suggesting that the excess tests were predominantly negative for *Cryptosporidium* during these two weeks (data not shown). The overall impact of this media messaging therefore appeared to have been a period of over-reporting likely including patients symptomatic for reasons unrelated to the BWN, who would not normally have sought advice from a healthcare service.

The impact of media coverage as a source of potential bias in syndromic surveillance has been reported infrequently. The nature of syndromic surveillance data collection renders these systems susceptible to shifts in healthcare seeking behaviour as a result of media coverage around a particular public health incident. We have previously reported the impact of media reporting on mumps clinician notifications illustrating potential bias in the public and health professionals [21]. The 2009 influenza A(H1N1) pandemic also generated intense media coverage and retrospective analysis of regional news coverage was suggested to influence the demand for local microbiological testing of samples for influenza A(H1N1) [22]. Conversely, media reporting can also be used as a useful source of information, including news outlets, discussion sites and disease reporting networks, to provide additional intelligence and increased awareness of public health issues, thus augmenting existing public health surveillance programmes [23].

In the context of the period of the BWN described here, understanding the surveillance data was critical to avoid misinterpretation and thus giving out inaccurate messages to healthcare professionals and the public. Considering the incubation period of cryptosporidiosis and the possible exposure of the population to the organism, the timing of the observed increases in syndromic indicators suggested a plausible increase in infections. The predominance of increases in diarrhoea and gastroenteritis indicators, and not of vomiting, was again in line with understood symptom presentation of cryptosporidiosis [3,4]. However, close working with front line local public health teams was important as this enabled all public health intelligence e.g. laboratory reporting to be included into the interpretation of syndromic data.

This paper highlights the real challenges and limitations of using symptom-based data for the identification of publicised outbreaks. We have shown an impact on health service providers in those areas affected by a BWN. This does not necessarily imply that there was an increase in the overall burden of gastroenteritis and diarrhoea in the community, just a change in healthcare seeking behaviour and therefore those cases registered by a medical practitioner. However, this represents an important message: during this event, despite the lack of confirmed cases there was a similar increase in the presentation of patients to health services, placing additional pressure on GPs, NHS 111, laboratories and possibly pharmacies for over-the-counter remedies. These increases were all likely resulting from the reporting of the possible public health risks through the media and resulted in a similar burden to some of these services as might be expected for a genuine incident. For future events, further work might need to focus on improved messaging from public health authorities. These messages need to balance the reassurance for patients that the public health interventions applied e.g. a BWN have reduced the risk of exposure to any potential hazards while also ensuring that exposed cases are identified. They also additionally need to alert local health service providers of the potential for increased burden during these periods.
